# Supplementary material for: Discovery of Nitro-azolo[1,5-a]pyrimidines with Anti-Inflammatory and Protective Activity against LPS-Induced Acute Lung Injury
Source: Pharmaceuticals (Basel). 2022 Apr 27;15(5):537. doi: 10.3390/ph15050537 (PMC9146423; doi:10.3390/ph15050537)
Supplement: Supplementary file 1 [file pharmaceuticals-15-00537-s001.zip › pharmaceuticals-1664402-supplementary.pdf]

Supporting Information

Discovery of nitro-azolo[1,5-*a*]pyrimidines with anti-inflammatory and protective activity against LPS-induced acute lung injury

Alexander Spasov [1], Vadim Kosolapov [1], Denis Babkov [1,*], Vladlen Klochkov [1], Elena Sokolova [1], Mikhail Miroshnikov [1], Alexander Borisov [1], Yulia Velikorodnaya [1], Alexey Smirnov [1], Konstantin Savateev [2], Victor Fedotov [2], Svetlana Kotovskaya [2] and Vladimir Rusinov [2]

[1] Department of Pharmacology & Bioinformatics, Scientific Center for Innovative Drugs, Volgograd State Medical University, Volgograd 400131 , Russia; aspasov@mail.ru (A.S.); vad-ak@mail.ru (V.K.);
klochkovvladlen@gmail.com (V.K.); sokolova210795@gmail.com (E.S.); mirwaisroman77@gmail.com (M.M.); borissow1978@rambler.ru (A.B.); alta-u@mail.ru (Y.V.); alexeysmirnov.volggmu@gmail.com (A.S.)

[2] Department of Organic and Biomolecular Chemistry, Ural Federal University Named after the First President of Russia B.N. Yeltsin, Mira Street, 19, Yekaterinburg 620002 , Russia; i-krafttt@yandex.ru (K.S.); vicww4@gmail.com (V.F.); sk-kotovskaya-665@yandex.ru (S.K.); v.l.rusinov@urfu.ru (V.R.)

* Correspondence: dababkov@volgmed.ru; Tel.: +7-9889608025

Table of contents

Spectra of compounds 2

Spectra of compounds

N-Isopropyl-5-methyl-6-nitrotetrazolo[1,5-*a*]pyrimidin-7-amine (9a)

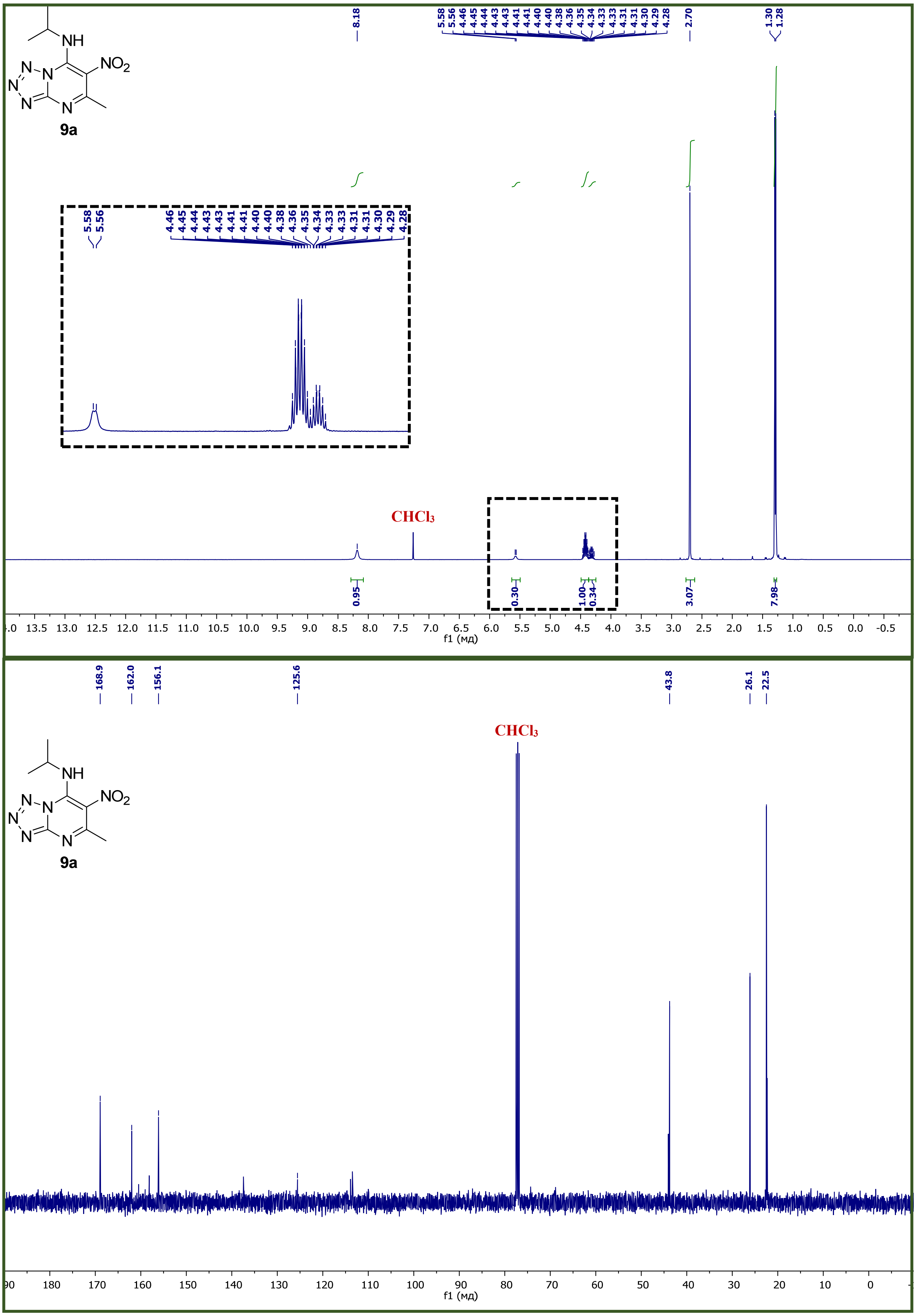

Figure S1. ^{1}H NMR (400 MHz, $CDCl_3$) and ^{13}C NMR (100 MHz, $CDCl_3$) spectra of **9a**

N-tert-Butyl-5-methyl-6-nitrotetrazolo[1,5-*a*]pyrimidin-7-amine (9b)

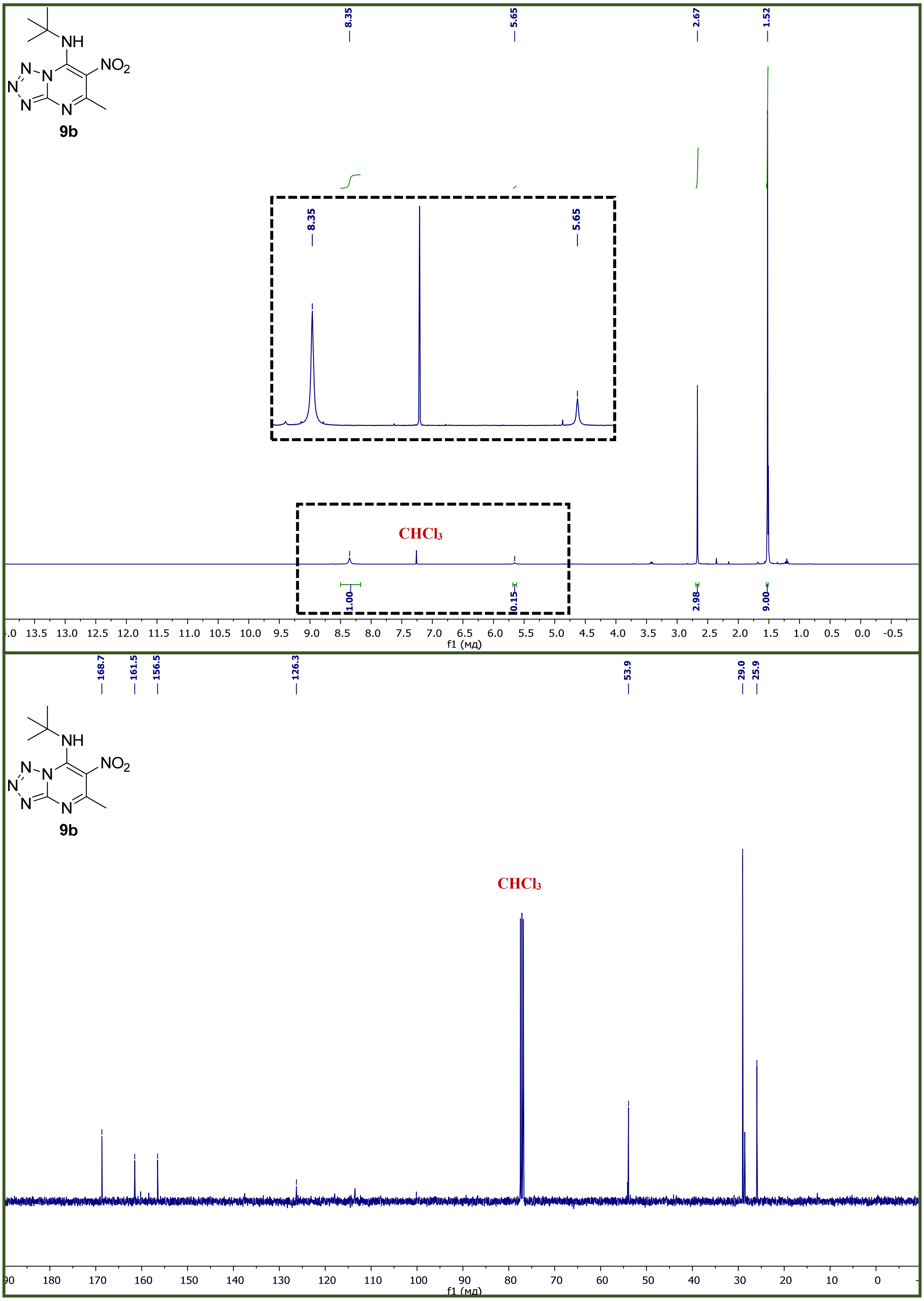

Figure S2. ^{1}H NMR (400 MHz, $CDCl_3$) and ^{13}C NMR (100 MHz, $CDCl_3$) spectra of **9b**

2-[(5-Methyl-6-nitrotetrazolo[1,5-*a*]pyrimidin-7-yl)-amino]ethanol (9c)

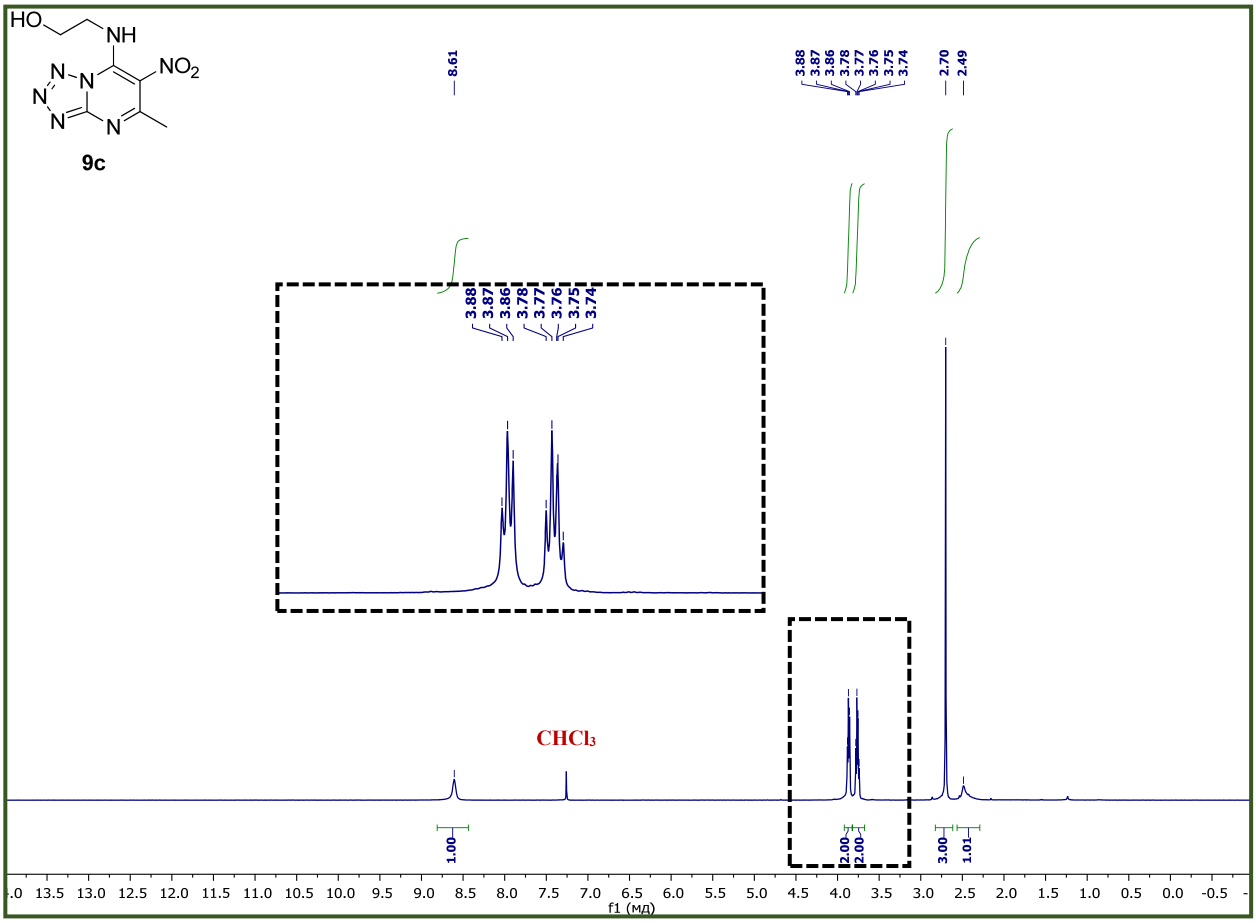

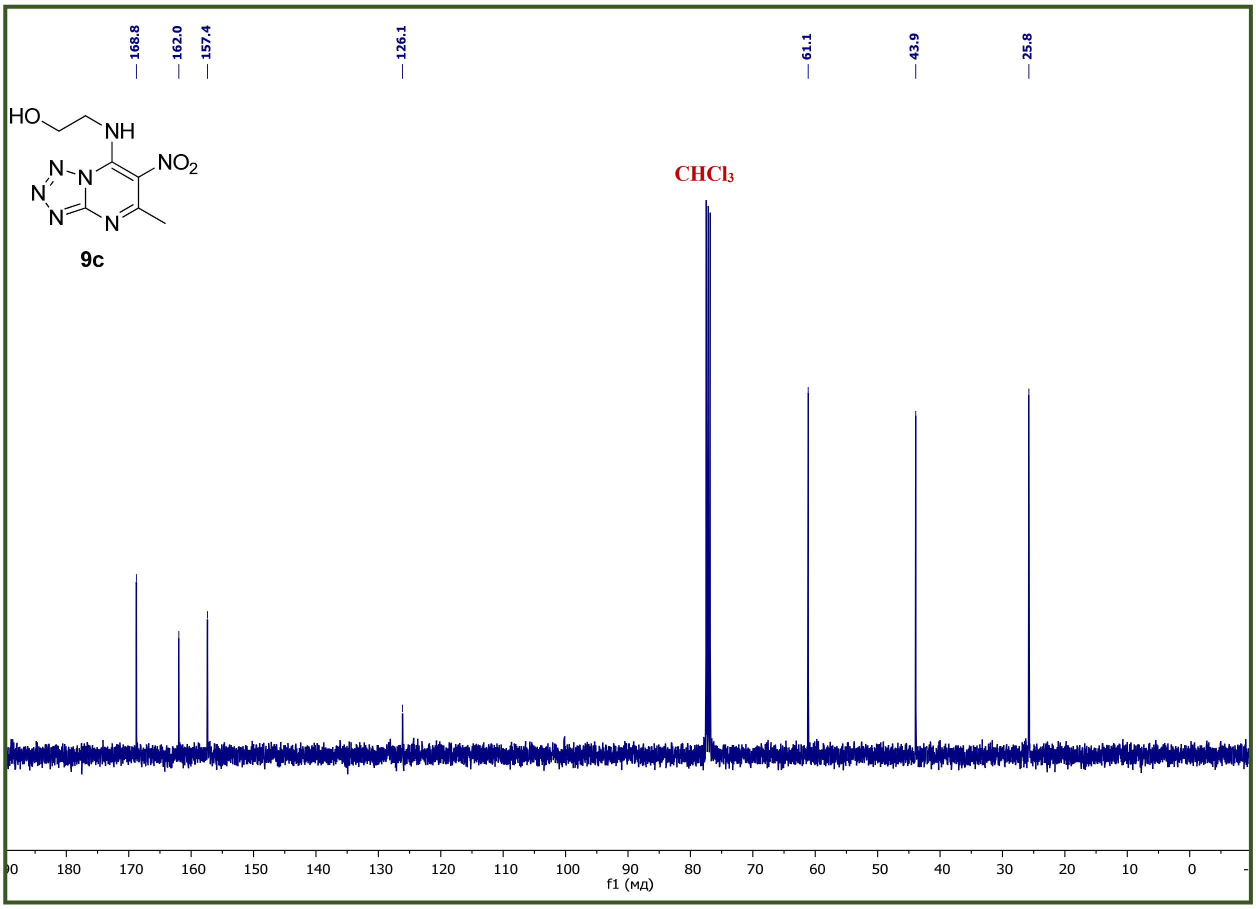

Figure S3. ^{1}H NMR (400 MHz, $CDCl_3$) and ^{13}C NMR (100 MHz, $CDCl_3$) spectra of **9c**

3-[(5-Methyl-6-nitrotetrazolo[1,5-*a*]pyrimidin-7-yl)-amino]propanol (9d)

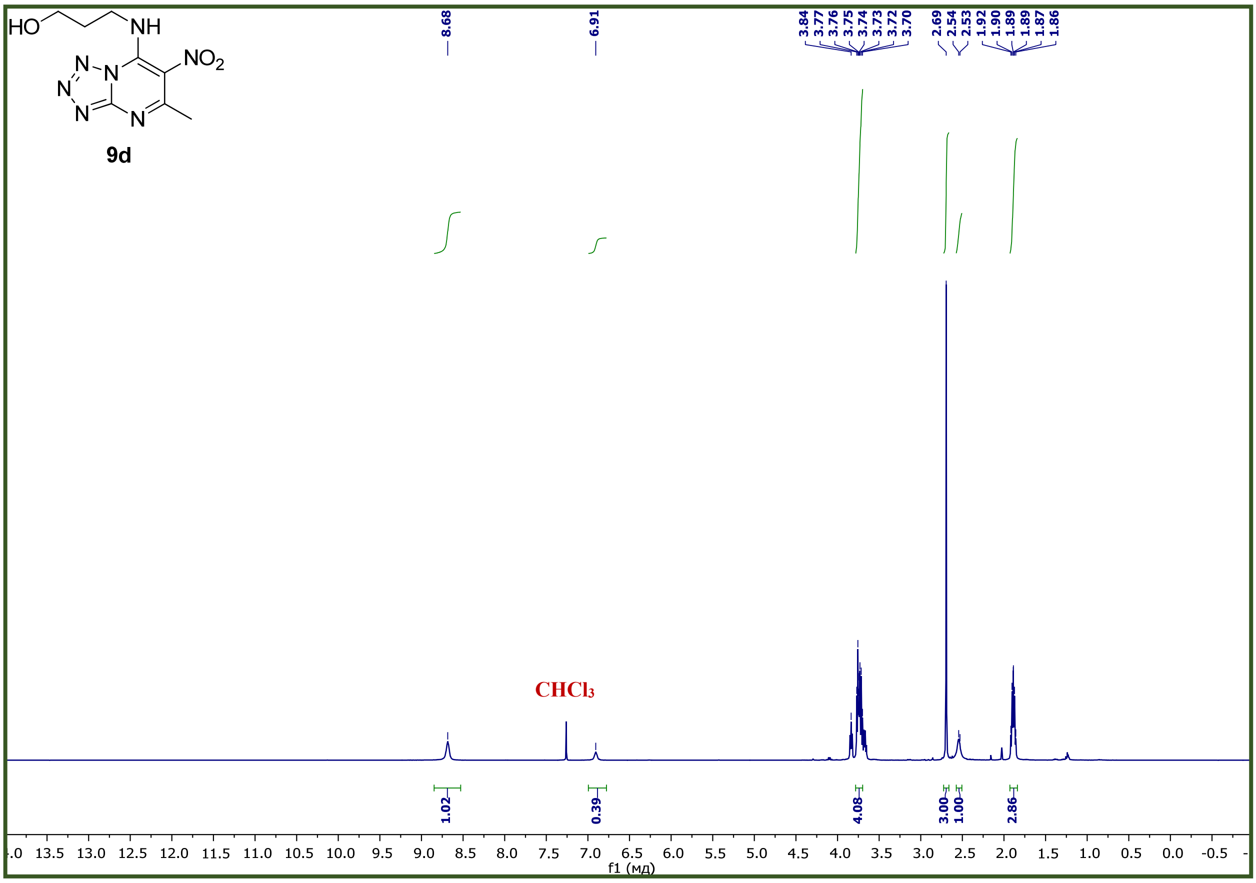

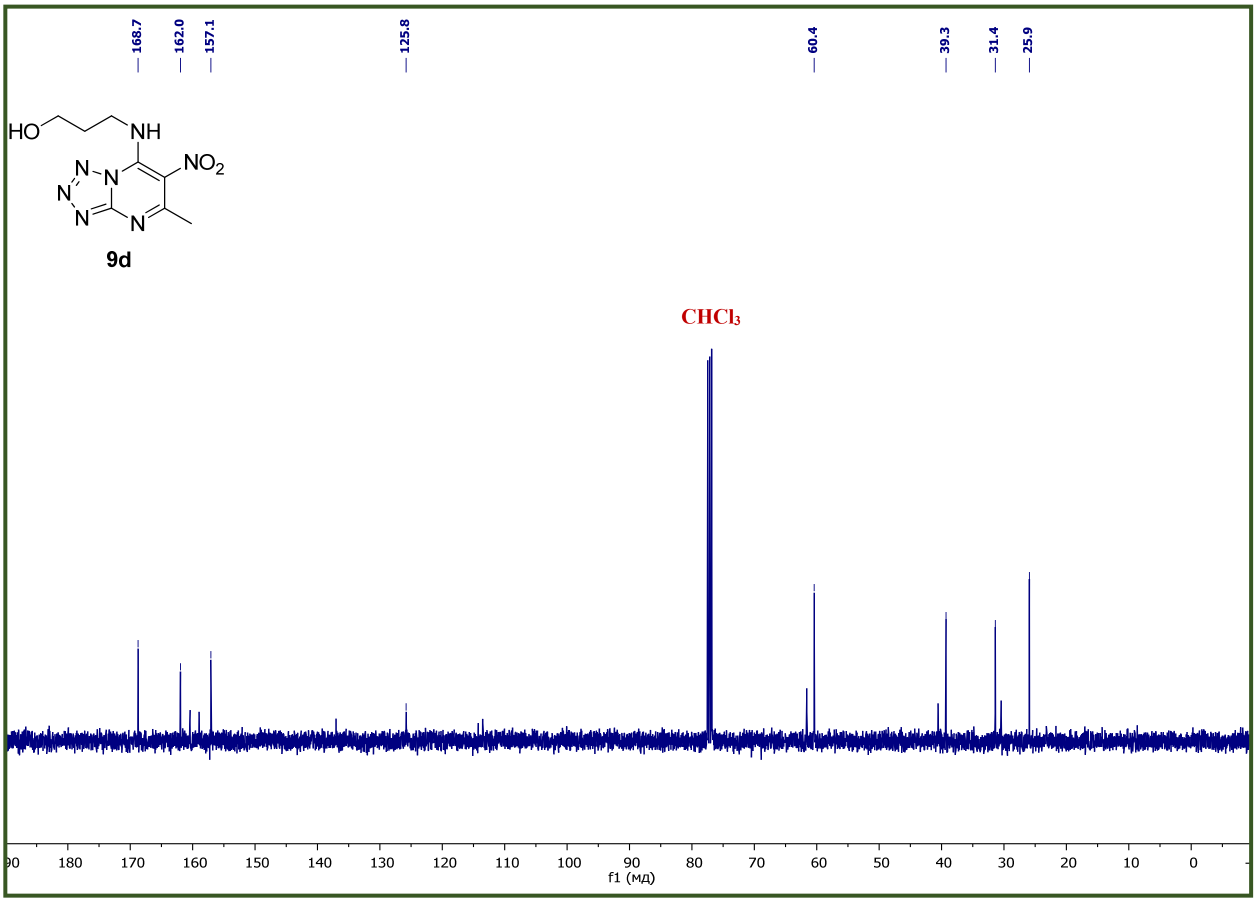

Figure S4. ^{1}H NMR (400 MHz, $CDCl_3$) and ^{13}C NMR (100 MHz, $CDCl_3$) spectra of **9d**

3-[(5-Methyl-6-nitrotetrazolo[1,5-*a*]pyrimidin-7-yl)-amino]propane-1,2-diol (9e)

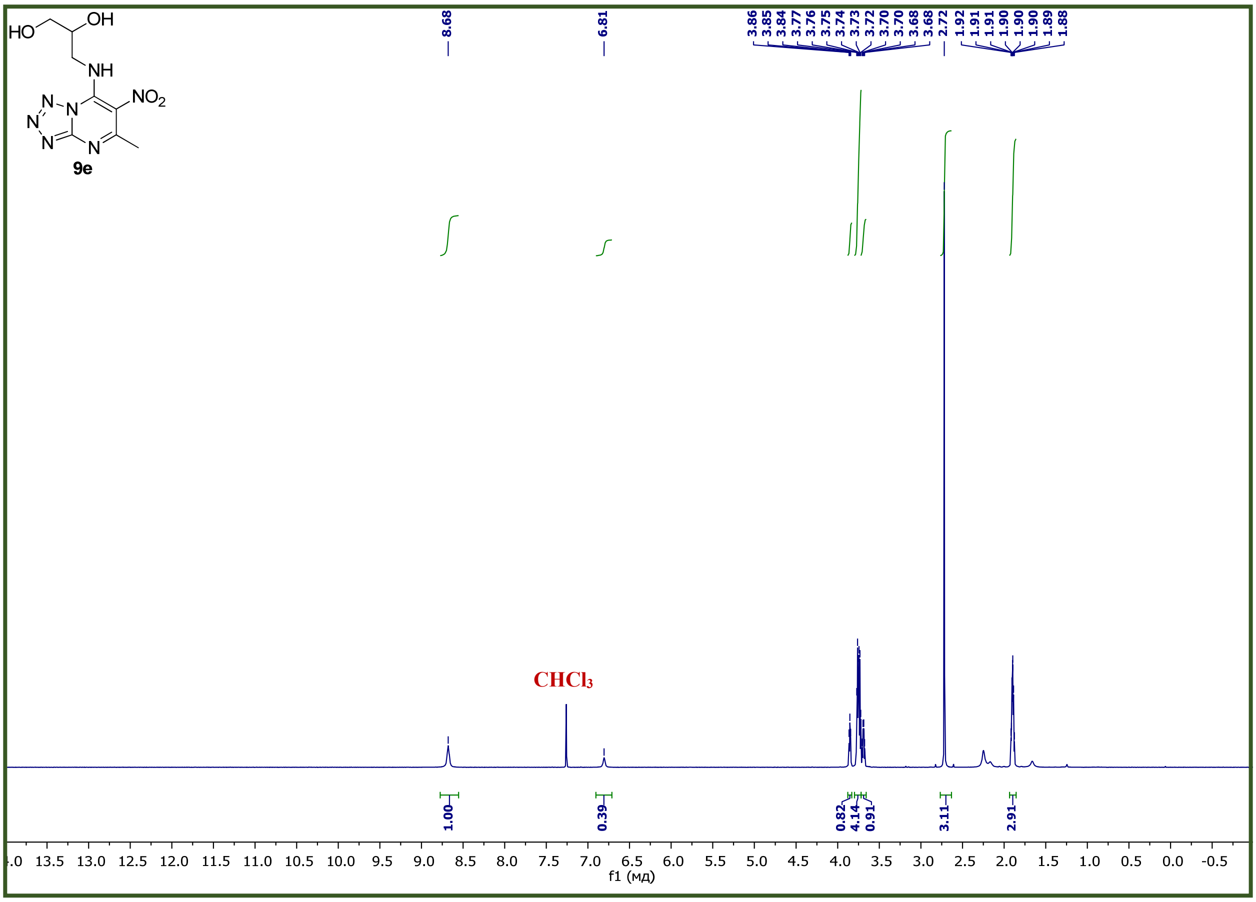

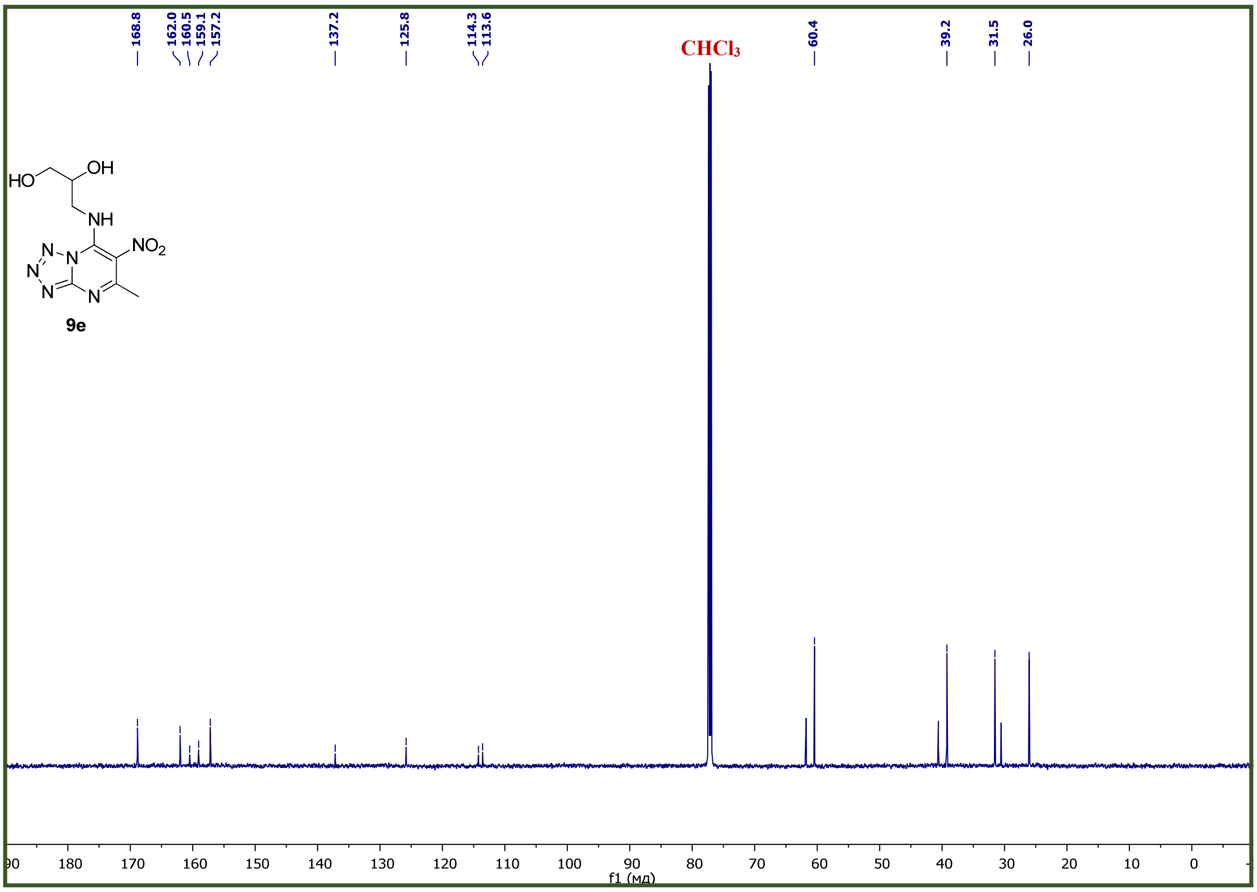

Figure S5. ^{1}H NMR (400 MHz, $CDCl_3$) and ^{13}C NMR (100 MHz, $CDCl_3$) spectra of **9e**

N-[2-(4-Chlorophenyl)ethyl]-5-methyl-6-nitrotetrazolo-[1,5-*a*]pyrimidin-7-amine (9f)

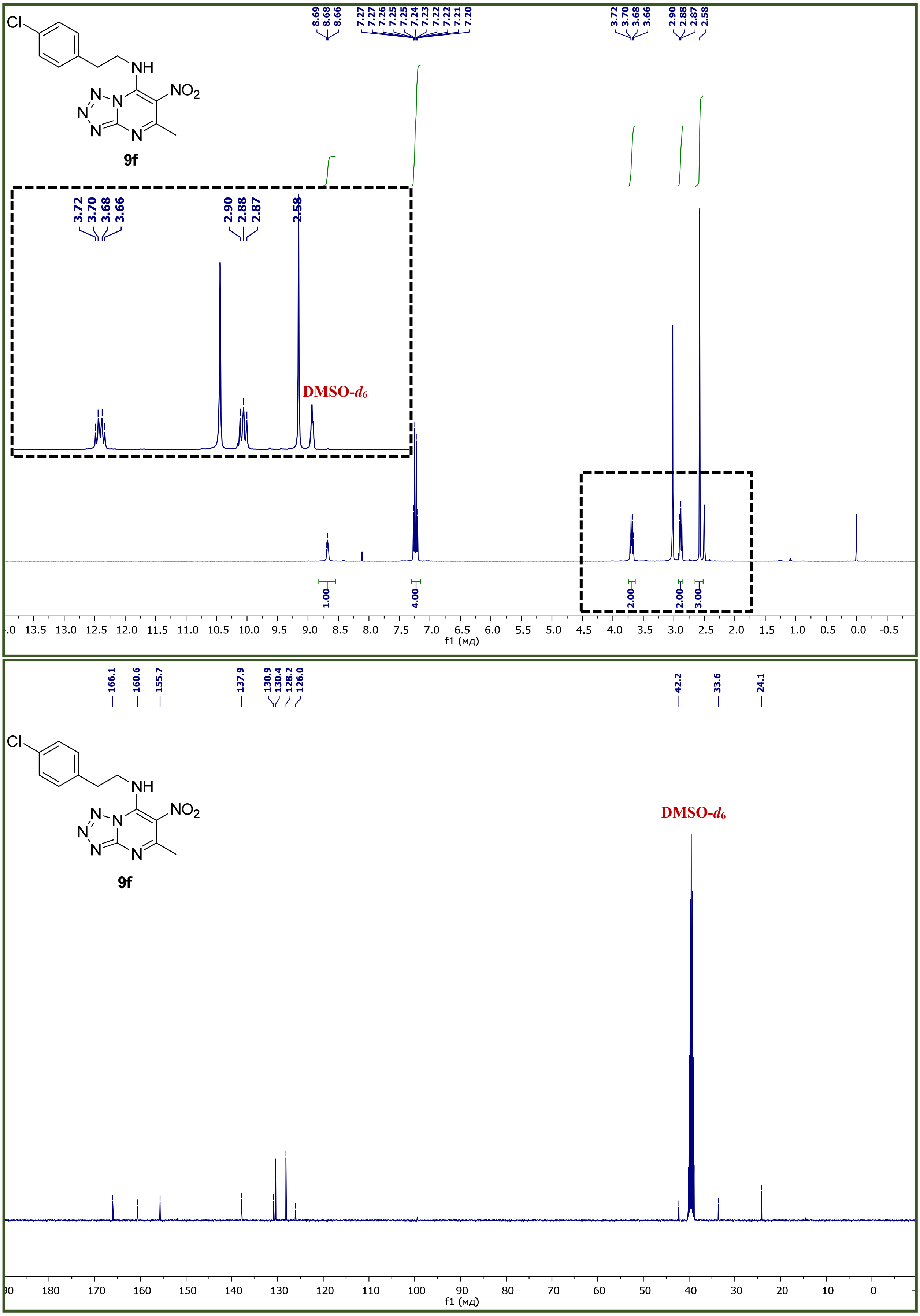

Figure S6. ^{1}H NMR (400 MHz, DMSO-d_6) and ^{13}C NMR (100 MHz, DMSO-d_6) spectra of **9f**

N-[2-(4-Hydroxyphenyl)ethyl]-5-methyl-6-nitrotetrazolo-[1,5-*a*]pyrimidin-7-amine (9g)

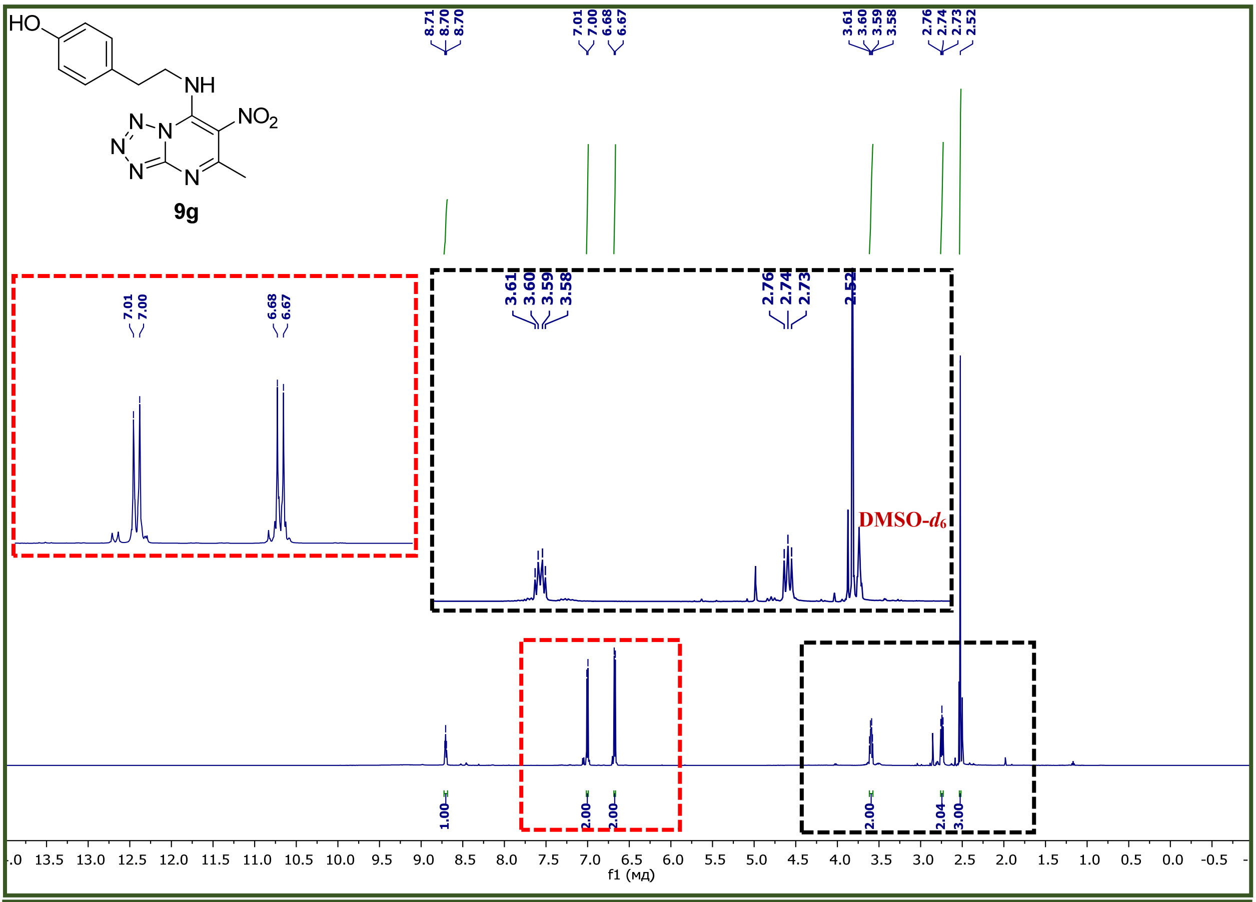

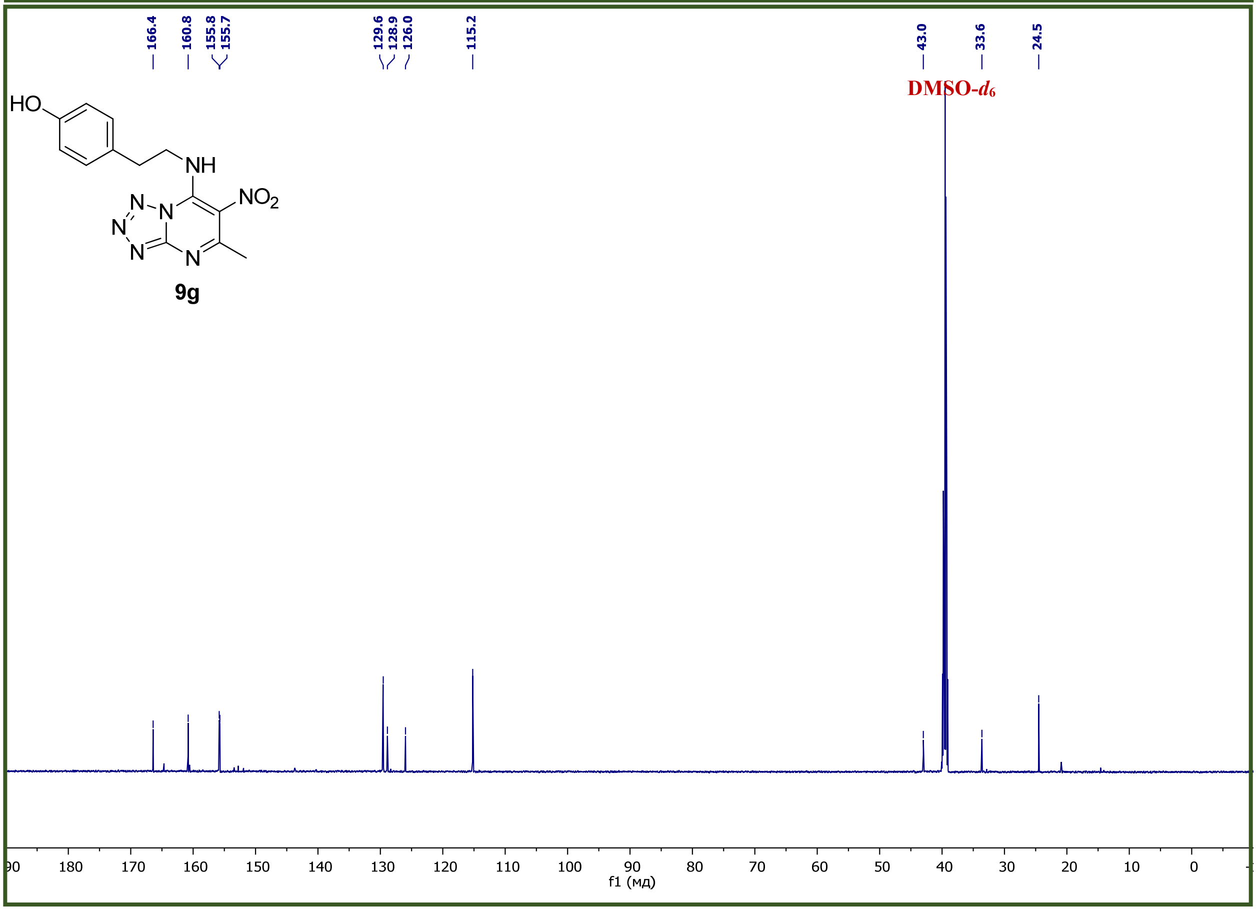

Figure S7. ^{1}H NMR (400 MHz, DMSO-d_6) and ^{13}C NMR (100 MHz, DMSO-d_6) spectra of **9f**

N-(4-Chlorophenethyl)-6-nitro-2-(prop-2-yn-1-ylsulfanyl)[1,2,4]triazolo[1,5-*a*]pyrimidin-7-amine (10f)

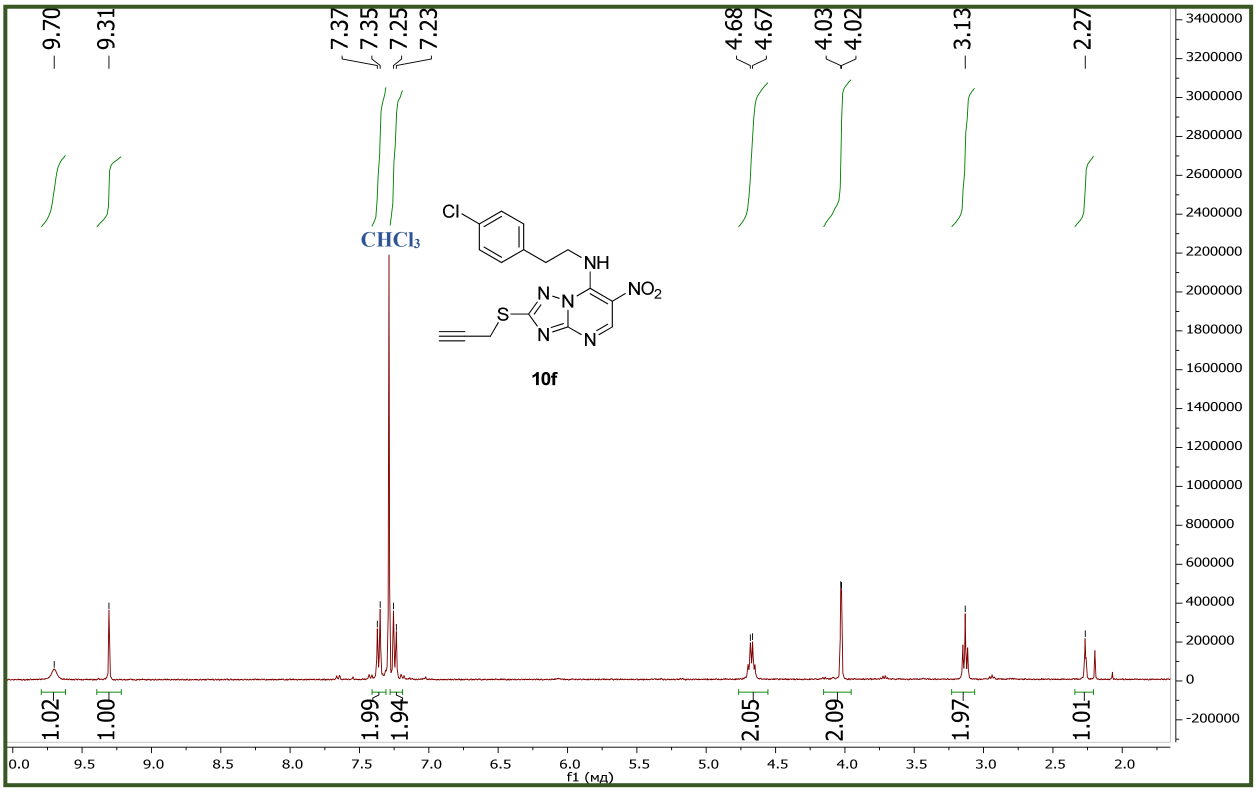

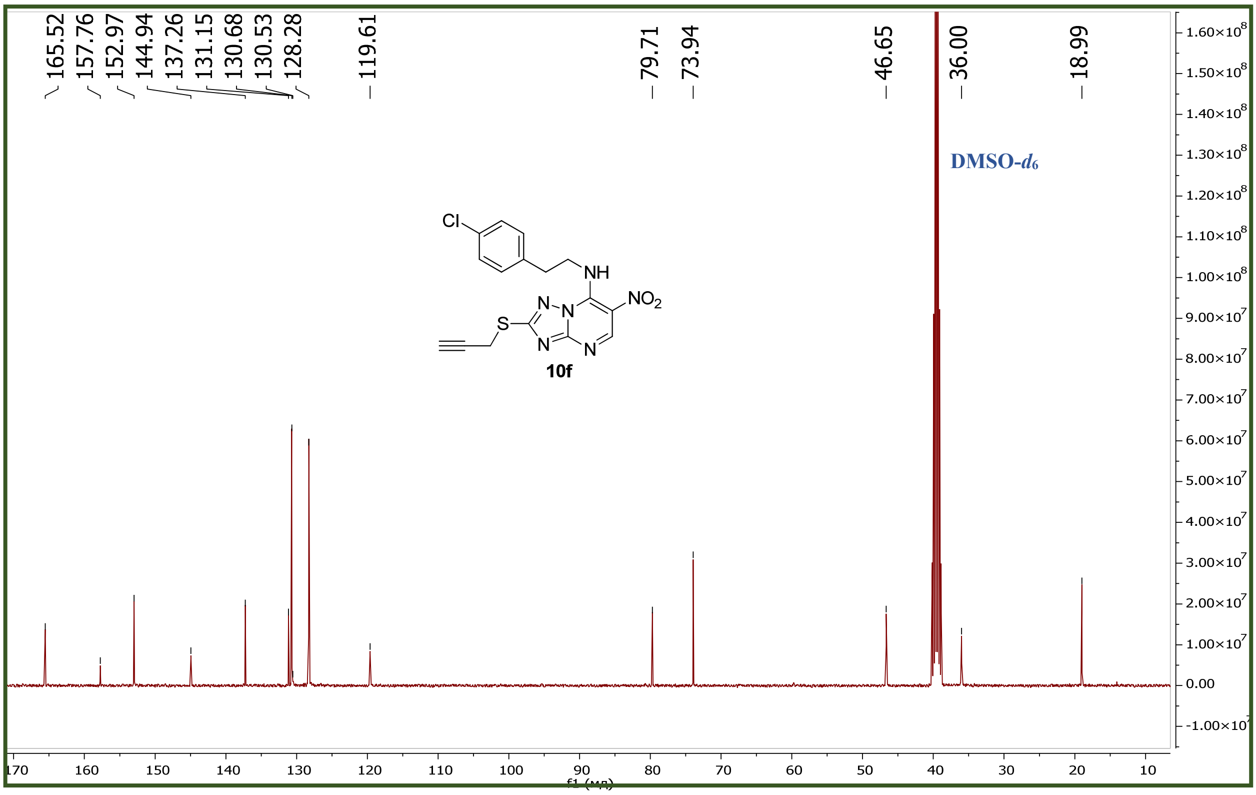

Figure S8. ^{1}H NMR (400 MHz, $CDCl_3$) and ^{13}C NMR (100 MHz, DMSO-$d_6$3) spectra of **10f**

4-{2-[(6-Nitro-2-(prop-2-yn-1-ylsulfanyl)[1,2,4]triazolo[1,5-*a*]pyrimidin-7-yl)amino]ethyl}phenol (10g)

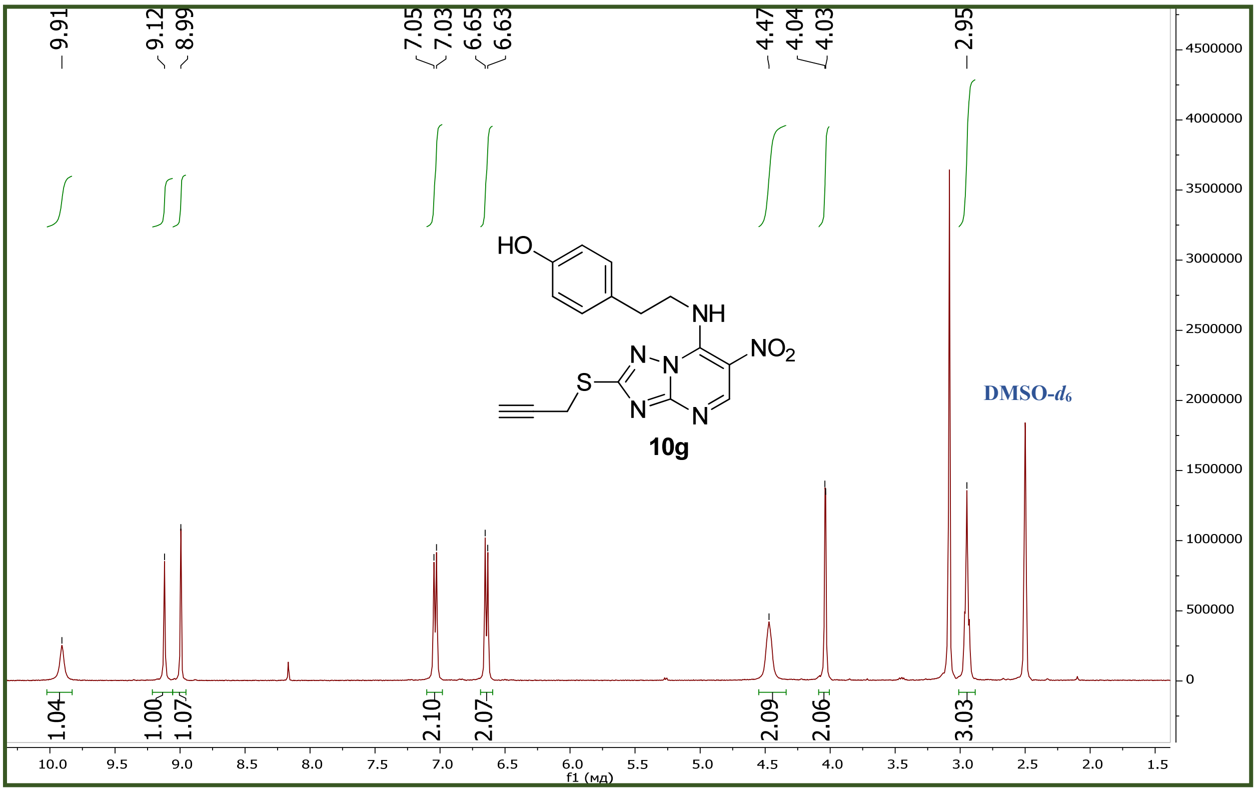

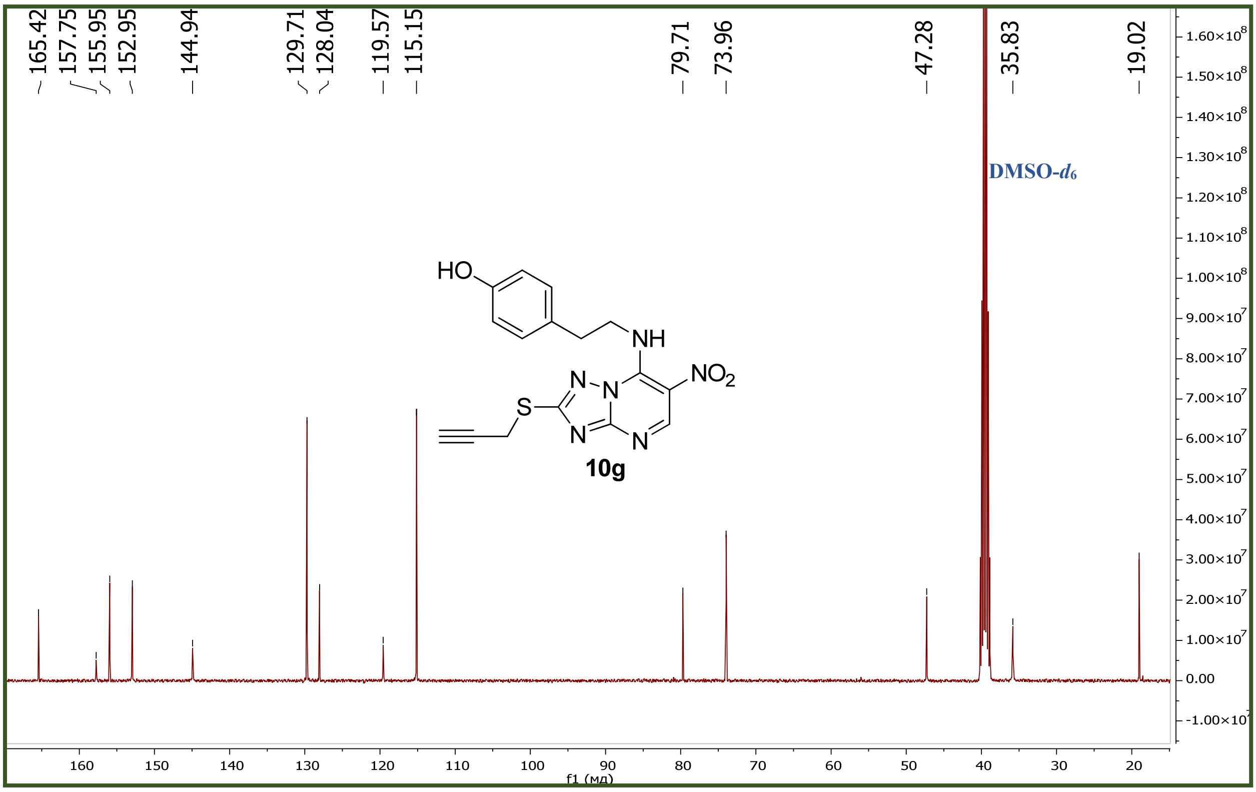

Figure S9. ^{1}H NMR (400 MHz, DMSO-*d*6) and ^{13}C NMR (100 MHz, DMSO-*d*6) spectra of **10g**

2-(5-Nitrofur-2-yl)-5-methyl-6-nitro-1,2,4-triazolo[1,5-*a*]pyrimidin-7-one aminoguanidinium salt (13)

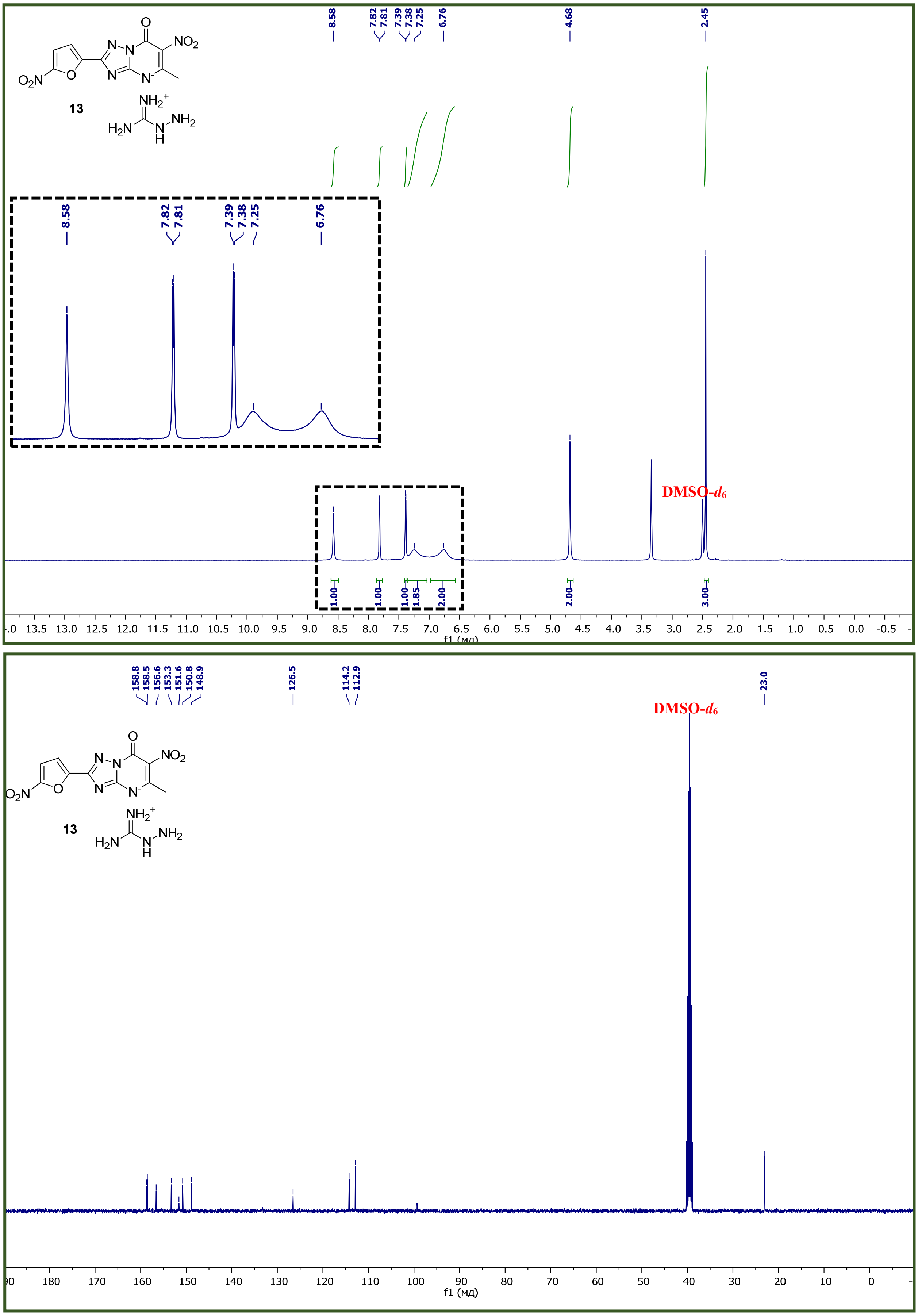

Figure S10. ^{1}H NMR (400 MHz, DMSO-d_6) and ^{13}C NMR (100 MHz, DMSO-d_6) spectra of **13**

2-(5-Nitrofur-2-yl)-5-methyl-6-nitro-1,2,4-triazolo[1,5-*a*]pyrimidin-7-one guanidinium salt (14)

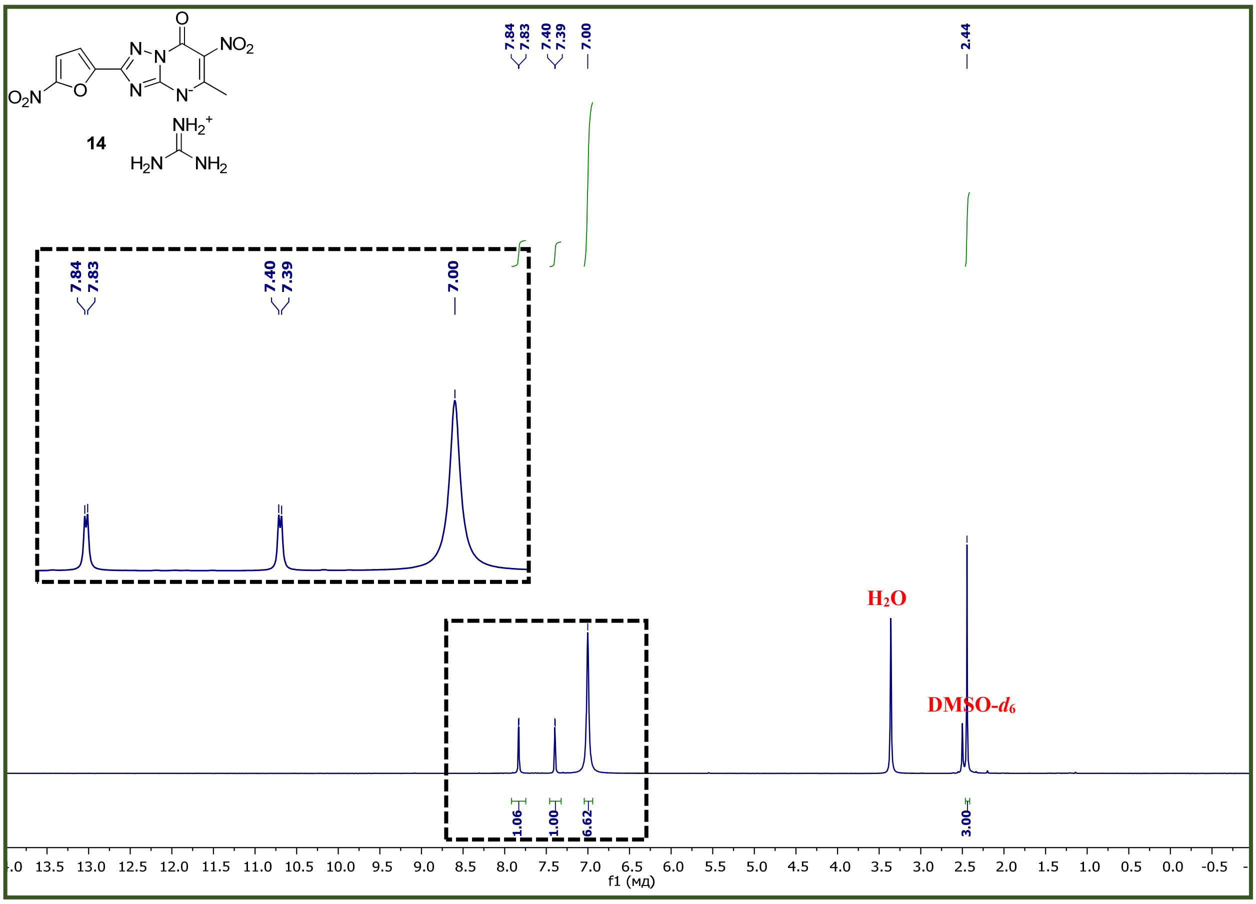

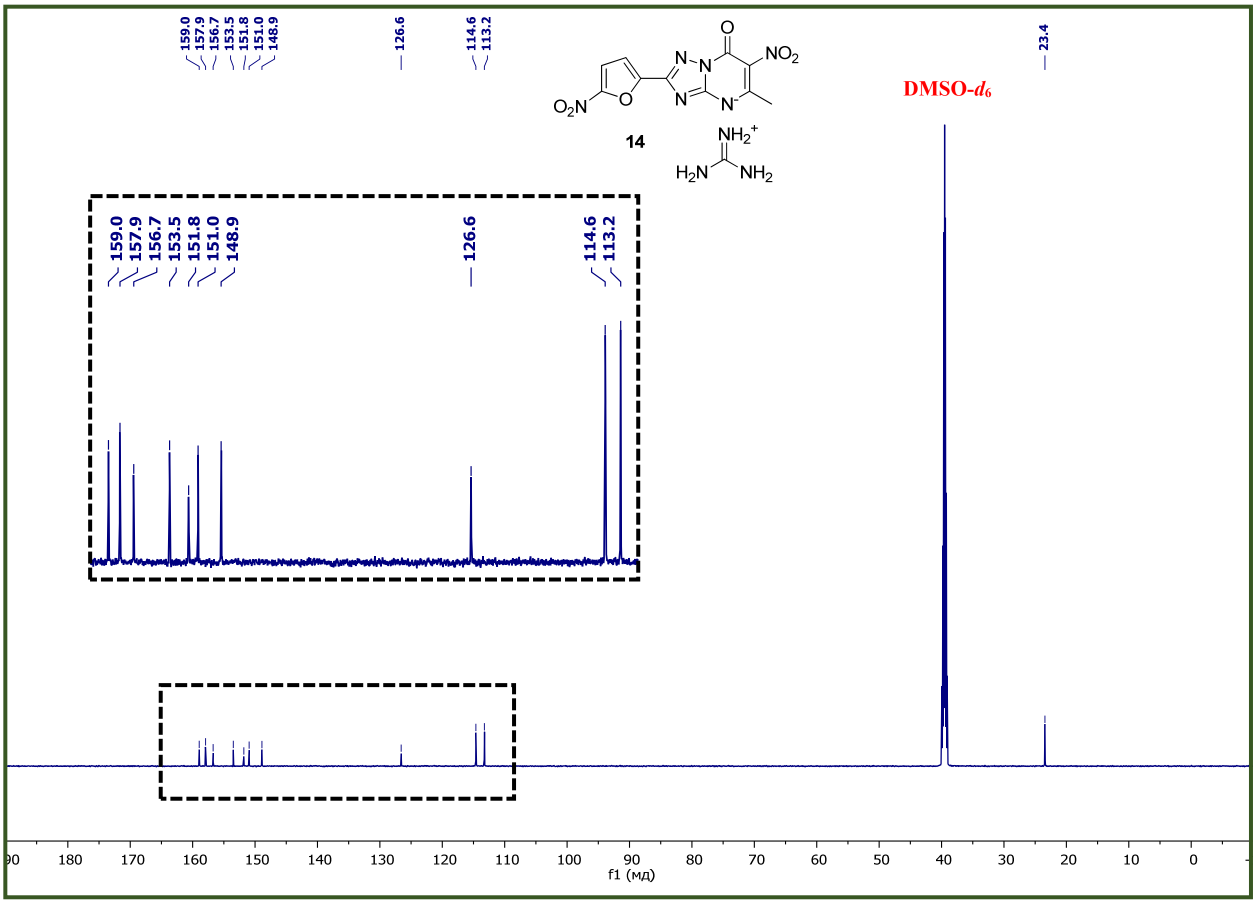

Figure S11. ^{1}H NMR (400 MHz, DMSO-d_6) and ^{13}C NMR (100 MHz, DMSO-d_6) spectra of **14**
